# Gastric malignant schwannoma presenting with upper gastrointestinal bleeding: a case report

**DOI:** 10.1186/1752-1947-6-37

**Published:** 2012-01-25

**Authors:** Masashi Takemura, Kayo Yoshida, Mamiko Takii, Katsunobu Sakurai, Akishige Kanazawa

**Affiliations:** 1 Department of Upper Gastrointestinal Surgery, Hyogo College of Medicine, 1-1, Mucogawa-machi, Nishinomiya City, Hyogo, 662-8501, Japan; 2 Department of Gastrointestinal Surgery, Osaka City General Hospital, 2-13-22, Miyakojima Hondori, Miyakojima, Osaka City, Osaka, 534-0021, Japan

**Keywords:** Gastric malignant schwannoma, S-100 protein, liver metastasis

## Abstract

**Introduction:**

We report a case of gastric malignant schwannoma presenting with gastrointestinal bleeding.

**Case presentation:**

A 70-year-old Japanese man presented with gastrointestinal bleeding to our hospital. Gastrointestinal endoscopy revealed a protruding lesion in the gastric body. Hematoxylin and eosin staining of biopsy specimens from this lesion revealed sheets of spindle cells. Immunohistochemistry revealed that these cells were positive for S-100 protein and negative for c-Kit and smooth muscle actin. Because mitosis was diffusely visible, this tumor was diagnosed as a gastric malignant schwannoma. Distal gastrectomy with lymph node dissection was performed and the patient's postoperative course was uneventful. However, five months after the surgery, he died from multiple liver metastases.

**Conclusion:**

Cases of gastric malignant schwannoma have rarely been reported. The efficacy of surgical resection and postoperative prognosis continues to remain unclear and should be investigated further.

## Introduction

Schwannomas are neurogenic tumors that originate from different organs as well as other areas throughout the body. Gastric schwannomas are rare; however, if they do occur in the gastrointestinal tract, the most common site is the stomach. Gastric schwannomas represent 0.2% of all gastric neoplasms [1]. Gastrointestinal endoscopy, such as endosonography, is the principal diagnostic tool for gastric schwannoma [2]. However, differentiating a schwannoma from other gastric submucosal tumors is often difficult. The definitive diagnosis of gastric schwannoma is established by pathological and immunohistochemical examination of resected surgical specimens [3].

Schwannomas are generally benign. Complete surgical removal is sufficient treatment for a benign gastric schwannoma because of excellent postoperative prognosis. Gastric malignant schwannomas are extremely rare and only a few cases have been reported [4,5]. We present the case of a 70-year-old man who presented with gastrointestinal bleeding because of gastric malignant schwannoma.

## Case presentation

**Figure 1:**
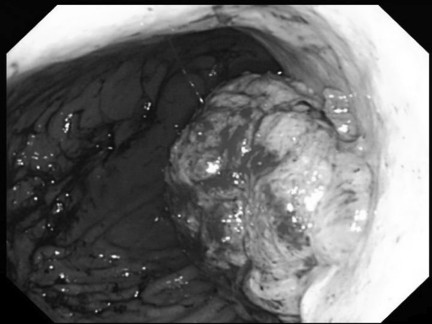
**Upper gastrointestinal endoscopy showing a protruding lesion at the lesser curvature of the gastric body**.

**Figure 2:**
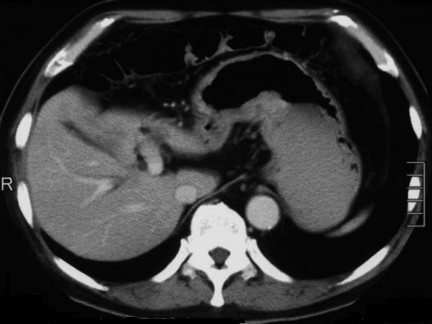
**Abdominal computed tomography showing a thickened posterior wall of the gastric body**. No evidence of lymph node swelling or a metastatic liver tumor is observed.

A 70-year-old Japanese man with melena that began five days before admission was admitted to our hospital. He complained of abdominal discomfort and epigastralgia. Five years before, he had undergone a right upper lobectomy for lung cancer (T1b N0 M0 Stage IA). Physical examination revealed no abnormal findings associated with the abdomen except a surgical scar on the right side of his chest. Evaluation of laboratory data on admission revealed that his hemoglobin level was 7.0 g/dL and hematocrit value was 23.1%. Upper gastrointestinal endoscopy revealed a distinctly protruding lesion (diameter: 5 cm) at the lesser curvature of the middle third of the gastric body (Figure 1). The surface of the tumor bled easily on contact with the endoscope. Microscopic examination of hematoxylin and eosin (H&E)-stained biopsy specimens of the lesion revealed sheets of spindle cells. Immunohistochemical studies showed that the tumor cells were positive for S-100 and negative for c-kit, CD34, and smooth muscle actin. The MIB-1 index was 48.5%. Based on these findings, the tumor was diagnosed as a gastric malignant schwannoma. Abdominal computed tomography (CT) revealed a thickened posterior wall of the gastric body (Figure 2). There was no evidence of lymph node swelling surrounding the stomach or metastatic liver tumors.

**Figure 3:**
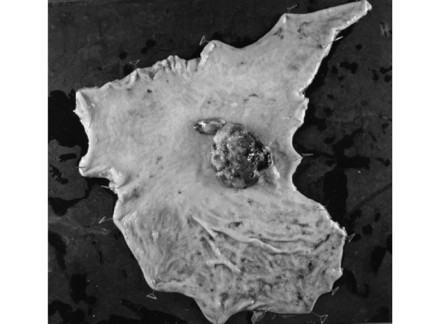
**Macroscopic findings of the resected specimens**. The elevated lesion approximately 6 × 5 cm in diameter is located at the lesser curvature of the gastric body.

**Figure 4:**
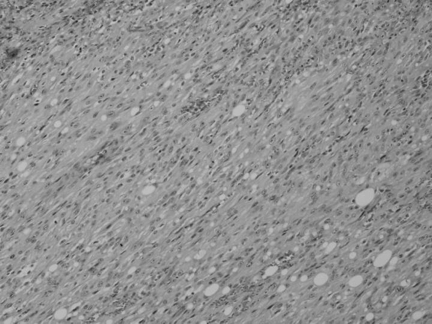
**Microscopic findings of the resected and hematoxylin and eosin-stained specimen showing a spindle cell neoplasm arranged in a palisade manner**.

**Figure 5:**
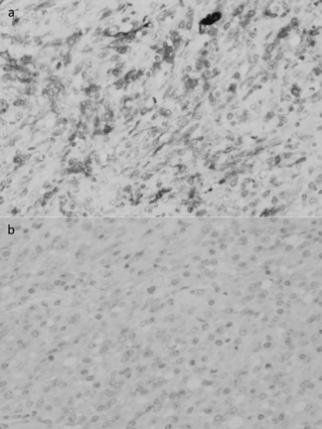
**Immunohistochemical analysis showing that the tumor cells were positive for S-100 protein (A), and negative for c-Kit (B) and smooth muscle actin**.

He underwent a distal gastrectomy with regional lymph node dissection. Macroscopically, the elevated lesion was approximately 6 × 5 cm in diameter and located at the lesser curvature of the gastric body (Figure 3). The covering mucosa was ulcerated. The tumor was located mainly in the proper muscle layer. There was no lymph node involvement and the surgical margin was negative for tumor cells. Microscopic examination of the resected and H&E-stained specimens showed a spindle cell neoplasm arranged in a palisade manner that was consistent with a schwannoma (Figure 4). Mitosis was scattered with 10 mitoses per 50 high-power fields. Immunohistochemistry revealed that the tumor cells were positive for S-100 protein and negative for c-kit and smooth muscle actin (Figure 5A,B). These histopathological and immunohistochemical findings are consistent with a gastric malignant schwannoma.

**Figure 6:**
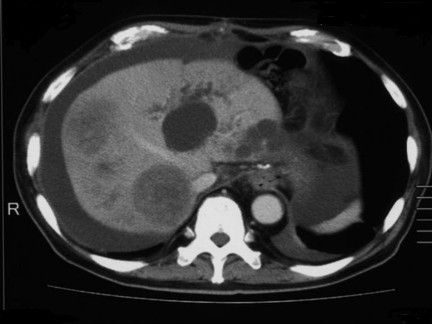
**Abdominal computed tomography three months after surgery showed multiple metastatic lesions in the liver and ascites**.

His postoperative course was uneventful and he was discharged from our hospital on day 12 after surgery. However, abdominal CT performed three months after surgery revealed multiple liver metastases and ascites (Figure 6).. He died five months after surgery without undergoing any additional treatment.

## Discussion

Schwannomas, also known as neurinomas and neurilemomas, are rare stromal tumors of spindle cells of the gastrointestinal tract that arise from the schwann cells of the gastrointestinal neural plexus. The stomach is the most common gastrointestinal site of schwannomas, which constitute 0.2% of gastric neoplasms [1,3]. Gastric schwannomas frequently occur in individuals 30 to 50 years old and are usually solitary lesions arising from the lesser curvature of the stomach [1,3,6].

Spindle cell tumors of the gastrointestinal tract are slow growing and covered with normal mucosa; they principally involve the submucosa and muscularis propria. The majority of spindle cell tumors of the gastrointestinal tract are believed to originate from the smooth muscle tissue. However, immunohistochemical staining has enabled differentiation of various spindle cell tumors [3]. Schwannomas can be distinguished from other spindle cell tumors of the stomach by S-100 protein staining. Tumor cells that are positive for S-100 protein and negative for smooth muscle actin, c-Kit and CD34 support the diagnosis of a schwannoma [7,8].

Preoperative differential diagnosis of gastric submucosal tumors is generally difficult. Computed tomography and gastrointestinal endoscopy are of limited use when attempting a definitive diagnosis of a gastric submucosal tumor [9]. Hoda et al. reported that endoscopic ultrasound (EUS)-guided sampling of gastrointestinal submucosal lesions is helpful [3]. They concluded that the diagnostic yield of EUS-fine needle aspiration (FNA) was 83.9% and that FNA can be used to establish a definite or probable diagnosis in the majority of patients with spindle cell tumors of the gastrointestinal tract. 18F-fluorodeoxyglucose positron emission tomography (FDG-PET) is a non-invasive diagnostic technique that enables quantification of tumor activity on the basis of altered tissue glucose metabolism. Recently, FDG-PET has been accepted as a powerful diagnostic tool for evaluating various tumors, even spindle cell tumors within the gastrointestinal tract [10-12]. Benz *et al. *demonstrated that FDG-PET can reliably discriminate malignant peripheral nerve sheath tumors from benign ones [10]. However, reports on the utility of FDG-PET for diagnosing gastric schwannomas are rare. Komatsu *et al. *reported a case of gastric schwannoma reflected by increased FDG uptake [12]. However, they showed that gastric schwannomas cannot be differentiated from gastrointestinal stromal tumors by FDG accumulation and that FDG-PET was not helpful for evaluating whether the tumors are benign or malignant. Therefore, further studies are required to clarify the utility of FDG-PET in the diagnosis of gastric schwannoma.

Bruneton *et al. *reviewed 112 cases of gastric schwannoma and found that the majority of the cases (63%) reported gastrointestinal bleeding as the first symptom and 42% presented with abdominal pain [13]. Gastrointestinal bleeding may be present in a case with deep ulceration due to gastric activity or ischemic changes in the covering mucosa and/or a schwannoma. The remaining patients had non-specific abdominal discomfort. In our patient who had gastrointestinal bleeding, the covering mucosa was found to be ulcerated during gastrointestinal endoscopy.

Gastric malignant schwannomas can be distinguished from benign schwannomas on the basis of histological examination of the resected specimens, not by clinical symptoms or imaging studies. The treatment strategy for gastric schwannoma is, therefore, the same regardless of whether the tumor is malignant or benign. Surgical resection is the only possible treatment for gastric schwannoma [1,14]. The postoperative prognosis for solitary benign schwannoma is excellent. Recurrent disease is generally associated with an incomplete surgical margin. Additionally, recent advances in molecular or target therapy have an important role for the treatment of gastrointestinal mesenchimal tumors. However, the usefulness of molecular therapy for gastric schwannoma is not clearly established because only a few cases with gastric schwannoma have been reported. On the other hand, Xabier *et al. *reported that there is a high expression rate of platelet-derived growth factor receptor (PDGFR) and c-kit in vestibular schwannoma [15]. They concluded that direct inhibition of these molecules by Gleevec^® ^(imatinib mesylate)may have relevant therapeutic applications for this kind of tumor. From these results, molecular therapy for gastric schwannoma may become an additional treatment modality and deserves further examination.

## Conclusions

Gastric malignant schwannomas are extremely rare, and only a few cases have been reported. Given the paucity of the currently available published literature, the efficacy of surgical resection and postoperative prognosis for such cases warrants further study.

## Consent

Written informed consent was obtained from the patient for publication of this case report and any accompanying images. A copy of the written consent is available for review by the Editor-in-Chief of this journal.

## Competing interests

The authors declare that they have no competing interests.

## Authors' contributions

All authors were actively involved in direct patient care and have read and approved the manuscript. MT is the principal author and was involved in the collection of data. AK contributed to writing the manuscript. MT, KY and KS were involved in the collection of relevant literature and proof read the manuscript.
